# Potential association between coronary artery disease and the inflammatory biomarker YKL-40 in asymptomatic patients with type 2 diabetes mellitus

**DOI:** 10.1186/1475-2840-11-84

**Published:** 2012-07-18

**Authors:** Hyun Min Kim, Byung-Wan Lee, Young-Mi Song, Won Jin Kim, Hyuk-Jae Chang, Dong-Hoon Choi, Hee Tae Yu, EunSeok Kang, Bong Soo Cha, Hyun Chul Lee

**Affiliations:** 1 Division of Endocrinology and Metabolism, Department of Internal Medicine, Yonsei University College of Medicine, Seoul, Korea; 2 Brain Korea 21 project for Medical Science, Yonsei University College of Medicine, Seoul, Korea; 3 Cardiology Division, Severence Cardiovascular Hospital, Yonsei University College of Medicine, Seoul, Korea; 4 Laboratory of Immunology and Infectious Diseases, Graduate School of Medical Science and Engineering, KAIST, Daejeon, Korea; 5 Department of Internal Medicine, Yonsei University College of Medicine, 250 Seongsanno, Seodaemun-gu, Seoul, 120-752, Korea

**Keywords:** YKL-40, Coronary artery disease, Type 2 diabetes

## Abstract

**Background:**

Inflammation plays an important role in coronary artery disease from the initiation of endothelial dysfunction to plaque formation to final rupture of the plaque. In this study, we investigated the potential pathophysiological and clinical relevance of novel cytokines secreted from various cells including adipocytes, endothelial cells, and inflammatory cells, in predicting coronary artery disease (CAD) in asymptomatic subjects with type 2 diabetes mellitus.

**Methods:**

We enrolled a total of 70 asymptomatic type 2 diabetic patients without a documented history of cardiovascular disease, and determined serum levels of chemerin, omentin-1, YKL-40, and sCD26. We performed coronary computed tomographic angiography (cCTA) in all subjects, and defined coronary artery stenosis ≥ 50 % as significant CAD in this study.

**Results:**

Subjects were classified into two groups: patients with suspected coronary artery stenosis on cCTA (group I, n = 41) and patients without any evidence of stenosis on cCTA (group II, n = 29). Group I showed significantly higher YLK-40 levels and lower HDL-C levels than group II (p = 0.038, 0.036, respectively). Levels of chemerin, omentin-1, and sCD26 were not significantly different between the two groups. Serum YKL-40 levels were positively correlated with systolic/diastolic BP, fasting/postprandial triglyceride levels, and Framingham risk score. Furthermore, YKL-40 levels showed moderate correlation with the degree of coronary artery stenosis and the coronary artery calcium score determined from cCTA. In multivariate logistic analysis, after adjusting for age, gender, smoking history, hypertension, and LDL-cholesterol, YLK-40 levels showed only borderline significance.

**Conclusions:**

YKL-40, which is secreted primarily from inflammatory cells, was associated with several CVD risk factors and was elevated in type 2 diabetic patients with suspected coronary artery stensosis on cCTA. These results suggest the possibility that the inflammatory biomarker YKL-40 might be associated with coronary artery disease in asymptomatic patients with type 2 diabetes mellitus.

## Background

Prediction and early diagnosis of coronary heart disease (CHD) facilitates appropriate intervention in the early stages of this disease. Despite our growing understanding of the pathophysiology of CHD in subjects with type 2 diabetes mellitus (T2D), predicting future CHD events remains largely reliant upon evaluation of traditional risk factors [1,2]. Even in apparently low- and intermediate-risk individuals assessed by conventional risk scores, a large number of diabetic subjects still experience cardiovascular disease (CVD) [3,4]. Therefore, novel biomarkers and predictive tools are required to facilitate optimal intervention in patients with coronary artery disease (CAD). An important feature of cardiometabolic disease is nonspecific local inflammatory processes that are accompanied by a low-grade systemic response [5]*.* Several studies in the past decade have investigated the effects of cytokines produced from different cells of origin on the progression of CAD in patients with type 2 diabetes to detect occult CAD. Of the clinical trials, the JUPITER trial of subjects with elevated high-sensitivity C-reactive protein (hsCRP) levels demonstrated how a biomarker can be used clinically to identify persons who might benefit from aggressive statin therapy [3]*.* Unfortunately, there was only a modest improvement in risk prediction when hsCRP was added to standard risk factor models.

In this study, we investigated the potential pathophysiological and clinical relevance of novel cytokines secreted from various cells including adipocytes, endothelial cells, and inflammatory cells in CAD prediction in asymptomatic subjects with type 2 diabetes mellitus. To do these, we selected four candidates biomarkers: chemerin, omentin-1, YKL-40, and sCD26, and validated subjects’ CAD status with coronary computed tomography (CT).

## Methods

### Study population

We cross-sectionally enrolled a total of 70 asymptomatic type 2 diabetic patients older than 45 years old attending the Diabetes Center of Severance Hospital. Exclusion criteria were as follows: (1) chest pain or equivalent symptoms according to the Rose questionnaire [6], (2) documented history of atherosclerotic vascular disease such as angina, myocardial infarction, cerebrovascular disease, or peripheral artery occlusive disease, (3) chronic kidney disease (creatinine > 1.5 mg/dL), (4) plasma aspartate transaminase (AST) and/or alanine transaminase (ALT) levels two-fold higher than the upper limit of normal, (5) hematological and/or malignant disease, and (6) previous history of radiocontrast-induced anaphylaxis. This study was approved by the Research Committee of Severance Hospital and written informed consent was obtained from all participants.

### Cardiac computed tomography protocol

Cardiac CT was performed using a 64-slice CT scanner (Sensation 64; Siemens Medical Solutions, Erlangen, Germany) as described previously [7]. Participants with a resting heart rate above 65 beats/min received an oral β-blocker (50 mg of metoprololtartate; Betaloc, Seoul, Korea) 1 hr before examination. In addition, a 0.3 mg sublingual dose of nitroglycerin was administrated just before scanning. Before multidetector coronary computed tomographic angiography (cCTA), a non-enhanced prospective ECG-gated sequential scan was performed to measure coronary artery calcification (CAC) under the following conditions: rotation time of 330 ms, slice collimation of 0.6 mm, slice width of 3.0 mm, tube voltage of 120 kV, tube current of 50 mAs, and table feed/scan of 18 mm. cCTA was performed using retrospective ECG-gating with the following scan parameters: rotation time of 330 ms, slice collimation of 64 × 0.6 mm, tube voltage of 100–120 kV, tube current of 600–800 mAs depending on the patient’s weight, table feed/scan of 3.8 mm, and pitch factor of 0.2. ECG-based tube current modulation was applied to 65 % of the R-R interval. A real-time bolus tracking method was used to trigger scan initiation. The mean radiation dose for the CAC scan and cCTA was 5.6 mSv (4.2–66.8 mSv) and 14.1 mSv (4.5–91.6 mSv), respectively. Contrast enhancement was achieved with 60 ml of Iopamidol (370 mg of iodine/ml, Iopamiro; Bracco, Milan, Italy) injected at 5 ml/s, followed by an injection of 30 ml of mixed saline (contrast agent: saline = 3: 7) and 30 ml of saline at 5 ml/s. A real-time bolus tracking method was used to trigger scan initiation. Two experienced radiologists prospectively and independently reviewed the cCTA images of the 70 patients. Differences in assessment were resolved by consensus. Each reader was blinded to the results of other examinations and clinical data.

### Risk factor analysis and laboratory assessments

We measured the patients’ height and weight to calculate their body-mass index (BMI). Blood pressure was measured twice with a mercury sphygmomanometer on the right upper arm after resting for at least 10 minutes in a seated position, and we used the average values of systolic and diastolic blood pressures. All individuals provided details regarding demographics, medical history, and medication profile. Subjects who were current or former smokers were defined as smokers. We investigated medication histories including use of insulin, oral hypoglycemic agents, statins, angiotensin receptor blockers (ARBs), angiotensin-converting enzyme (ACE) inhibitors, or calcium channel blockers. Any individual with high total cholesterol (>200 mg/dl), high low-density lipoprotein cholesterol (LDL-cholesterol) (>160 mg/dl), low high-density lipoprotein cholesterol (HDL-cholesterol) (<35 mg/dl for men, <40 mg/dl for women), and/or high triglycerides (>150 mg/dL), or current use of lipid-lowering therapy were coded as having dyslipidemia. Hypertension was defined as a self-reported history of hypertension and/or a history of antihypertensive medication use or a systolic blood pressure of 140/90 mmHg once or more at the time of the visit.

Blood samples were collected from subjects after an overnight fast. All participants underwent laboratory analyses, including determination of fasting plasma glucose levels by the glucose oxidase method, total cholesterol, triglyceride, HDL-cholesterol, LDL-cholesterol, blood urea nitrogen, creatinine, AST, and ALT using a routine Hitachi 7600 autoanalyzer (Hitachi Instruments Service, Tokyo, Japan), glycated hemoglobin (HbA1c) by high-performance liquid chromatography (HPLC) using a Variant II Turbo system (Bio-Rad Laboratories, Hercules, CA), glycated albumin (GA) by an albumin-specific proteinase assay, ketamine oxidase, albumin assay reagents (LUCICA GA-L, Asahi Kasei Pharma Co., Tokyo, Japan) using a Hitachi 7699 Pmoduleautoanalyzer, and hsCRP by a latex-enhanced immunoephelometric method using a BN II analyzer (Dade Behring, Newarkand, DE). Serum insulin levels were measured in duplicate using an immunoradiometric assay (IRMA) method (Beckman Coulter, Fullerton, CA). Insulin resistance was assessed by using the homeostasis model assessment – insulin resistance (HOMA_IR_) equation as follows: HOMA_IR_ = (fasting insulin (in microunits per milliliter) × fasting serum glucose (in millimoles per liter)/22.5). Blood samples were collected 120 min after food intake for glucose and triglyceride analyses.

The separated sera were stored at −70 °C until chemerin (Millipore, Bedford, MA), omentin-1 (Millipore, Bedford, MA), YKL-40 (Quidel, San Diego, CA), and sCD26 (Bender MedSystems, Vienna, Austria) levels could be measured using commercially available ELISA kits according to the manufacturers’ instructions. The intra- and inter-assay variation was 5.0 % and 4.0 % for the chemerin ELISA, 1.5 % and 5.8 % for the omentin-1 ELISA, 6.0 % and 6.6 % for the YKL-40 ELISA, and 4.6 % and 9.1 % for the sCD26 ELISA, respectively.

### Statistical analysis

All statistical analyses were performed with PASW statistics software (version 18.0; SPSS Inc., Chicago, IL). Continuous variables with a normal distribution are expressed as means ± SD unless otherwise indicated. A *P* value < 0.05 was considered statistically significant. Intergroup differences in baseline characteristics were analyzed by independent t-tests or the chi-square test. Differences among three groups according to the severity of stenosis were tested using one-way ANOVA and subsequent post hoc analyses (Tukey's HSD). Pearson’s correlation coefficients were calculated to examine the relationships between serum YKL-40 levels and metabolic and cardiovascular variables. Multivariate logistic regression analysis was used to determine the factors associated with suspected CAD on cCTA in patients with diabetes.

## Results

A total of 70 subjects (28 men and 42 women; mean age 59.6 ± 6.3 years) were enrolled in this study. The mean duration of diabetes was 7.2 ± 1.2 years. The levels of serum HbA1c and GA were 7.29 ± 1.22, and 19.2 ± 6.1 %, respectively. The mean BMI of the study subjects was 26.0 ± 3.4 kg/m^2^.

As outlined in the Duke coronary artery index [8,9], we defined the degree of stenosis as significant stenosis (≥ 50 %), minimal stenosis (< 50 %), or normal based on the lumen diameter of diseased vessels. According to the degree of stenosis on cCTA, we divided the patients into two groups: those with suspected CAD (n = 41) and those without any evidence of CAD (n = 29). And we consulted with a cardiologist about the patients with significant CAD (≥ 50 % stenosis), and invasive coronary angiography (ICA) was recommended in 10 patients who showed radiological and clinical signs of CAD. Nine patients eventually underwent ICA and five patients received revascularization (one coronary artery bypass graft and four percutaneous coronary interventions).

The baseline clinical characteristics of the group of type 2 diabetic patients with suspected CAD and those with no evidence of CAD are shown in Table 1. There were no significant differences between the two groups in age, gender, BMI, diabetes duration, glucose parameters including fasting/postprandial glucose, HbA1c, GA, and HOMA_IR_, lipid profiles or hsCRP levels. Systolic blood pressure tended to be higher in the subjects with suspected CAD compared to those with no evidence of CAD, although it did not reach a statistical significance (131 ± 18 *vs.* 122 ± 17 mmHg, p = 0.066). There were no significant differences between the two groups in conventional coronary risk factors such as prevalence of hypertension, dyslipidemia, family history of premature CAD, smoking history, or Framingham risk score (FRS). The history of medication use including insulin, oral hypoglycemic agents, statins, and anti-hypertensive agents was similar in both groups. However, the coronary artery calcium score (CACS) was much higher in patients with CAD than those without CAD (206 ± 342 *vs.* 3 ± 13, *p* = 0.003).

**Table 1:** Baseline characteristics of the study participants

	**Suspected CAD (n = 41)**	**No evidence of CAD (n = 29)**	**p-value**
Age (years)	60 ± 5	58 ± 6	0.237
Male (n,%)	19 (46.3)	9 (31.0)	0.149
Body-mass index (kg/m^2^)	25.7 ± 3.3	26.4 ± 3.6	0.468
SBP (mmHg)	131 ± 18	122 ± 17	0.066
DBP(mmHg)	74 ± 10	71 ± 8	0.305
Diabetes duration (years)	9.1 ± 7.1	8.7 ± 6.1	0.813
Hypertension (n,%)	29 (70.7)	19 (65.5)	0.418
Dyslipidemia (n,%)	18 (62.1)	27 (65.9)	0.47
Family history of CAD (n,%)	25 (61.0)	17 (58.6)	0.519
Smoking Hx (n,%)			
Never smoker	29 (70.7)	25 (86.2)	
Ex-smoker	4 (9.8)	3 (10.3)	
Current smoker	8 (19.5)	1 (3.4)	
FPG (mg/dL)	132 ± 46	136 ± 41	0.718
PPG-2 hrs (mg/dL)	219 ± 104	196 ± 90	0.331
HbA1c (%)	7.49 ± 1.24	7.14 ± 1.19	0.398
GA (%)	19.7 ± 6.8	18.4 ± 4.9	0.362
hsCRP (mg/dL)	2.75 ± 5.39	1.83 ± 2.23	0.335
Total cholesterol (mg/dL)	175 ± 50	166 ± 43	0.414
LDL cholesterol (mg/dL)	100 ± 37	94 ± 39	0.585
HDL cholesterol (mg/dL)	45 ± 11	46 ± 12	0.745
TG (mg/dL)	130 ± 76	118 ± 44	0.41
Postprandial TG (mg/dL)	124 ± 60	112 ± 43	0.352
HOMA_IR_	5.25 ± 6.51	4.18 ± 3.10	0.364
Framingham risk score (FRS)	7.3 ± 5.6	5.2 ± 6.3	0.18
CACS	206 ± 342	3 ± 13	0.003
Chemerin (ng/mL)	201.3 ± 82.3	193.1 ± 75.9	0.674
Omentin (ng/mL)	104.4 ± 29.4	106.4 ± 23.5	0.767
YKL-40 (ng/mL)	148.6 ± 82.3	96.7 ± 73.0	0.013
sCD26 (ng/mL)	381.5 ± 399.4	332.0 ± 188.9	0.494
Insulin (%)	12 (29.3)	9 (31.0)	0.54
Sulfonylurea (%)	18 (43.9)	14 (48.3)	0.453
Metformin (%)	29 (70.7)	24 (82.8)	0.192
Thiazolidinedione (%)	12 (29.3)	7 (24.1)	0.423
ARB or ACE inhibitors (%)	26 (63.4)	12 (41.4)	0.057
Calcium channer blockers (%)	10 (24.4)	6 (20.7)	0.474
Beta blockers (%)	5 (12.2)	2 (6.9)	0.381
Statins (%)	22 (53.7)	15 (51.7)	0.533

*DM*, diabetes mellitus; *CAD*, coronary artery disease; *SBP*, systolic blood pressure; *DBP*, diastolic blood pressure; *FPG*, fasting plasma glucose; *PPG-2 hrs*, postprandial plasma glucose-2 hrs; *HbA1c*, glycated hemoglobin; *GA*, glycated albumin; *hsCRP*, high-sensitive C-reactive protein; *LDL*, ligh-density lipoprotein; *HDL*, high-density lipoprotein; *TG*, triglycerides; *HOMA*_*IR*_, homeostasis model assessment– insulin resistance; *CACS*, coronary artery calcium score; *ARB*, angiotensin receptor blocker; *ACE*, angiotensin converting enzyme.

**Figure 1:**
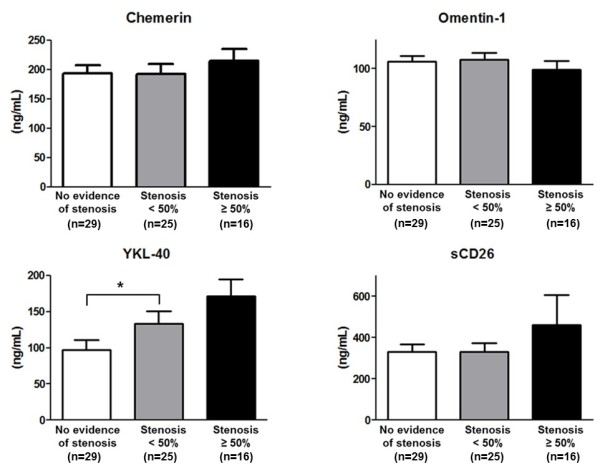
Serum levels of chemerin, omentin-1, YKL-40, and sCD26 according to the severity of coronary artery stenosis on cCTA in patients with type 2 diabetes mellitus.

The serum levels of novel candidate cytokines were evaluated. Of the four candidates, only YKL-40 showed a statistical difference between the two groups (148.6 ± 82.3 *vs.* 96.7 ± 73.0 ng/mL, *p* = 0.013). The other candidate biomarkers had comparable levels in the two groups (chemerin 201.3 ± 82.3 *vs.* 193.1 ± 75.9 ng/mL, omentin-1 104.4 ± 29.4 *vs.* 106.4 ± 23.5 ng/mL, and sCD26 381.5 ± 399.4 *vs.* 332.0 ± 188.9 ng/mL, *p* = 0.674, *p* = 0.767, and *p* = 0.494, respectively). The levels of cytokines were further classified according to the degree of coronary artery stenosis on cCTA (Figure 1). This sub-analysis revealed that serum YKL-40 levels were higher in patients with coronary artery stenosis than patients with no evidence of stenosis, and were further increased in patients with stenosis ≥ 50 % (96.7 ± 73.0 *vs.* 133.5 ± 81.9 *vs.* 171.3 ± 94.7 ng/mL, *p* = 0.017). Serum chemerin and omentin-1 levels were similar among 3 groups (*p* = 0.584, *p* = 0.637, respectively). sCD26 levels tended to be higher in patients with ≥ 50 % stenosis compared to patients with no evidence of stenosis or < 50 % stenosis; however, these differences were not statistically significant (459.2 ± 589.5 *vs.* 332.0 ± 188.9 ng/mL, 459.2 ± 589.5 *vs.* 331.7 ± 205.3 ng/mL, *p* = 0.44, *p* = 0.453, respectively).

Next, we evaluated the correlation between serum YKL-40 levels and various metabolic and cardiovascular parameters (Table 2). There were significant but weak correlations between YKL-40 concentrations, systolic/diastolic blood pressure, and fasting/postprandial triglyceride levels. YKL-40 levels were positively correlated with several cardiovascular parameters including Framingham risk score, degree of coronary artery stenosis on cCTA, and CACS on cardiac CT. However, serum YKL-40 concentrations did not correlate significantly with fasting insulin, LDL-cholesterol, HDL-cholesterol, hsCRP levels, BMI, or HOMA_IR_.

**Table 2:** Metabolic and cardiovascular parameters and their correlations with serum YKL-40 levels

	**Correlation coefficient**	**P-value**
SBP (mmHg)	0.269	0.017
DBP (mmHg)	0.287	0.011
Fasting triglycerides (mg/dL)	0.234	0.039
Postprandial triglycerides (mg/dL)	0.386	< 0.001
Framingham risk score (FRS)	0.289	0.01
Degree of coronary artery stenosis on cCTA (%)	0.407	0.001
CACS	0.407	< 0.001

*SBP*, systolic blood pressure; *DBP*, diastolic blood pressure; *TG*, triglycerides; *cCTA*; coronary computed tomographic angiography; *CACS*, coronary artery calcium score.

To determine the factors that were predictive of coronary artery stenosis in the subjects with asymptomatic type 2 diabetes, we performed univariate and multivariate logistic analyses. Three statistical models with different independent variables were used. We entered non-modifiable and modifiable risk variables in models 1 and 2, respectively. Serum YKL-40 showed only borderline significance after adjusting for age, gender, smoking history, hypertension, and LDL-cholesterol level. After adjustment for hsCRP, YKL-40 still showed borderline significance for predicting suspected CAD in patients with type 2 diabetes mellitus (Table 3).

**Table 3:** Univariate and multivariate logistic regression analyses for suspected CAD on cCTA in type 2 diabetic patients

	**Odds ratio (95 % CI)**	**p-value**
Univariate	1.009 (1.001 - 1.016)	0.018
Multivariate		
Model 1*	1.007 (1.000 - 1.015)	0.047
Model 2†	1.007 (1.000 - 1.015)	0.054
Model 3‡	1.008 (1.000 - 1.015)	0.048

*CAD*, coronary artery disease; *cCTA*; coronary computed tomographic angiography; *CI*, confidence interval.

* Adjusted for age and gender.

† Adjusted for age, gender, smoking history, hypertension, and low density lipoprotein cholesterol.

‡ Adjusted for age, gender, smoking history, hypertension, low density lipoprotein cholesterol and high-sensitive C-reactive protein.

## Discussion

In contrast to the characteristics of type 1 diabetes mellitus, an etiologic chronic proinflammatory state is thought to be involved in the pathophysiology of T2D, ultimately resulting in atherosclerotic changes [10]. Due to recent advances in understanding circulating molecular actions, cross-talk between adipose tissue, the immune system, and the vascular wall in T2D, several novel biomarkers and predictive tools to aid in the appropriate intervention of CAD have been proposed [11]. Adipocytes enriched by pathological accumulation or *de novo* synthesis secrete a host of mediators called adipokines. Adipokines produced in obesity regulate inflammation by both overproduction of proinflammatoryadipokines and lower production of anti-inflammatory adipokines, and regulate vascular tone by producing vasorelaxants and vasoconstrictors [12]. In addition, cytokines secreted from inflammatory or endothelial cells play a key role in the inter-organ crosstalk between metabolic disease and vascular disease.

In this study, we investigated the clinical relevance of novel biomarkers secreted from adipocytes, inflammatory cells, and/or endothelial cells in Korean subjects with T2D. We performed cardiac CT and adopted cCTA to validate the presence of CAD. There were three main findings. First, compared to type 2 diabetic patients with no evidence of CAD on cCTA, the level of serum YKL-40, which is secreted mainly by inflammatory cells, was markedly increased in subjects with suspected CAD. Second, serum YKL-40 levels were positively correlated with several metabolic and cardiovascular parameters. Third, after adjusting for other cardiovascular risk factors and hsCRP, which is a traditional inflammatory marker, YKL-40 showed some borderline significance for predicting any degree of coronary artery stenosis in subjects with T2D.

YKL-40 has been implicated in previous studies in diabetes and atherosclerosis. Inflammation is known to be a contributing factor to T2D, and the numbers of inflammatory cells within the visceral adipose tissue are increased. Several recent studies have documented that YKL-40 levels were elevated in both type 1 and type 2 diabetic subjects independent of BMI [13,14]. Consistent with other studies, we found a positive correlation between YKL-40 levels and several metabolic parameters, such as fasting/postprandial triglyceride levels in this study.

In the initiation and progression of atherosclerosis, activated macrophages take up lipids and then these lipid-rich macrophages secret inflammatory mediators that stimulate VSMC migration and proliferation [15], resulting in atherosclerosis. It is known that YKL-40 is secreted by activated macrophages and neutrophils in different tissues with inflammation, vascular smooth muscle cells (VSMC), cancer cells, and arthritic chondrocytes [16]. In this regard, YKL-40 may be involved in the early stage of atherosclerosis and CAD. So we categorized the asymptomatic type 2 diabetic patients into two groups – those with any degree of stenosis in coronary artery on cCTA (suspected CAD) and those with completely normal coronary artery on cCTA (no evidence of CAD). In our study, we did not find any significant association between suspected CAD and hsCRP, the most widely used and evaluated inflammatory biomarker for cardiovascular diseases. YKL-40 levels, however, were significantly higher in patients with suspected CAD than those with no evidence of CAD. YKL-40 is produced locally by inflammatory cells, however, hsCRP is produced by hepatocyte in response to high circulating cytokine levels. Taken together, YKL-40 might be more sensitive marker than hsCRP for predicting suspected CAD in type 2 diabetic patients. Because few studies have been conducted on YKL-40 as a novel marker for predicting occult CAD in asymptomatic subjects with normal glucose tolerance or diabetes mellitus, our results make a novel contribution to the clinical relevance of YKL-40 in asymptomatic type 2 diabetic patients.

There is a controversy in the association between serum YKL-40 level and the severity of atherosclerosis. In our study, serum YKL-40 level was correlated with the degree of coronary artery stenosis on cCTA, and increased along with the degree of stenosis. However, recent study concluded that circulating YKL-40 was not specifically related to the size of stenosis [17]. Another study investigating the role of YKL-40 in patients with peripheral arterial disease showed that severity of atherosclerosis is associated with higher YKL-40 levels [18]. These conflicting results may be due to differences in the study participants and diagnostic modality for evaluation of coronary artery and severity of atherosclerosis. Understanding the action mechanism of YKL-40 in atherosclerosis could help clarify the clinical meaning of serum YKL-40.

We investigated chemerin in this study as it is a proinflammatoryadipokine that modulates chemotaxis and activates macrophages [19], thereby facilitating the development of atherosclerosis and CAD [20,21]. Also, we chose to evaluate omentin-1 as it is an anti-inflammatory molecule [22]. Yoo*et al.* showed that circulating omentin-1 levels were decreased in patients with T2D compared to normal glucose controls, and independently correlated with arterial stiffness and carotid plaque after adjusting for other risk factors in type 2 diabetic patients [23]. However, the serum levels of chemerin and omentin-1 were not significantly associated with the severity of coronary artery stenosis in this study (Figure 1). Both markers are secreted from adipocytes, and epicardial adipose tissue may have direct effects on coronary artery atherosclerosis due to its close proximity to coronary artery [24]. So, locally produced adipokines by EAT rather than circulating level might affect the process of atherosclerosis. Previous study has shown that the mRNA and protein expressions of chemerin were significantly higher in epicardial adipose tissue from patients with CAD compared to that from normal control, however, there was no significant differences in circulating chemerin level between two groups [25]. In addition, adipose tissues are known to secret several adipokines that have an important role in the initiation of insulin resistance [26] or endothelial dysfunction [27], measurement of visceral adipose mass and levels of other adipokines, such as adiponectin or leptin, could help elucidate why these two markers did not predict CAD in our study [28,29].

sCD26 is a dipeptidyl peptidase-4 (DPP-4) that is ubiquitously expressed in epithelial and endothelial cells and that circulates in plasma [30]. The main source of this enzyme is thought to be microvascular endothelial cells [31]. Although a recent study demonstrated that sCD26 might impair insulin sensitivity in both an autocrine and paracrine fashion, and has a role in linking adipose tissue and metabolic syndrome [32], few clinical studies have examined the association between sCD26 levels and endothelial dysfunction or atherosclerosis. In our study, sCD26 levels tended to increase in patients with significant CAD (coronary artery stenosis ≥ 50 % on cCTA). Although this finding was not statistically significant in our study, it is possible that increased sCD26 levels may be associated with the progression of atherosclerosis.

Further studies are required to validate our findings due to several limitations of our study. First, we only investigated a relatively small number of cases. Second, because of the inherent weaknesses of cross-sectional design, we could not determine if there was a causal relationship between YKL-40 and the initiation and progression of CAD. Lastly, we did not measure the levels of the novel markers we examined in age-matched controls or in normal glucose-tolerant subjects with CAD. However, serum YKL-40 levels have been shown to be higher in patients with type 1 and type 2 diabetes mellitus than control subjects with normal glucose tolerance [13,14].

## Conclusions

In conclusion, we evaluated whether four novel cytokines (chemerin, omentin-1, YKL-40, and sCD26) secreted from different origins in type 2 diabetic patients were associated with CAD, and found that only serum YKL-40 levels were markedly elevated in patients with type 2 diabetes patients with suspected CAD on cCTA compared to patients with no evidence of CAD. Furthermore, YKL-40 levels were positively correlated with the severity of CAD and various metabolic parameters. However, after adjusting for other cardiovascular risk factors, YKL-40 showed only borderline significance. Based on these results, we suggest the possibility that the inflammatory biomarker YKL-40 might be associated with coronary artery disease in asymptomatic patients with type 2 diabetes mellitus. However, further large-scale clinical studies are warranted.

## Abbreviations

CHD, Coronary heart disease; T2D, Type 2 diabetes mellitus; CVD, Cardiovascular diseases; CAD, Coronary artery disease; hsCRP, High-sensitivity C-reactive protein; CT, Computed tomography; AST, Aspartate transaminase; ALT, Alanine transaminase; cCTA, Coronary computed tomographic angiography; CAC, Coronary artery calcification; BMI, Body-mass index; ARBs, Angiotensin receptor blockers; ACEi, Angiotensin-converting enzyme inhibitors (ACEi); LDL, Low-density lipoprotein; HDL, High-density lipoprotein; HbAlc, Glycated hemoglobin, GA, Glycated albumin; HOMA-IR, Homeostasis model assessment of insulin resistance (HOMA-IR); ICA, Invasive coronary angiography; FRS, Framingham risk score; CACS, Coronary artery calcium score.

## Competing interests

The authors have no conflict of interest to declare.

## Authors’ contributions

HMK analyzed data and wrote the manuscript. BWL designed research and reviewed/edited the manuscript. WJK collected data. YMS performed the biochemical analysis. HJC contributed discussion. DHC contributed discussion. HTY contributed discussion. ESK contributed discussion. BSC contributed discussion. HCL contributed discussion. All authors read and approved the final manuscript.
